# Proteomic datasets of HeLa and SiHa cell lines acquired by DDA-PASEF and diaPASEF

**DOI:** 10.1016/j.dib.2022.107919

**Published:** 2022-02-04

**Authors:** Zelu Huang, Weijia Kong, Bertrand Jernhan Wong, Huanhuan Gao, Tiannan Guo, Xianming Liu, Xiaoxian Du, Limsoon Wong, Wilson Wen Bin Goh

**Affiliations:** a School of Chemical and Biomedical Engineering, Nanyang Technological University, Singapore; b School of Biological Sciences, Nanyang Technological University, Singapore; c Department of Computer Science, National University of Singapore, Singapore; d Zhejiang Provincial Laboratory of Life Sciences and Biomedicine, Key Laboratory of Structural Biology of Zhejiang Province, School of Life Sciences, Westlake University, Zhejiang, China; e Institute of Basic Medical Sciences, Westlake Institute for Advanced Study, Zhejiang, China; f Bruker (Beijing) Scientific Technology Co., Ltd, Shanghai, China; g Lee Kong Chian School of Medicine, Nanyang Technological University, Singapore

**Keywords:** DDA, DIA, PASEF, HeLa, SiHa, DDA, Data Dependent Acquisition, DIA, Data Independent Acquisition, MS, Mass Spectrometry, PASEF, Parallel Accumulation Serial Fragmentation

## Abstract

We present four datasets on proteomics profiling of HeLa and SiHa cell lines associated with the research described in the paper “PROTREC: A probability-based approach for recovering missing proteins based on biological networks” [1]. Proteins in each cell line were acquired by two different data acquisition methods. The first was Data Dependent Acquisition-Parallel Accumulation Serial Fragmentation (DDA-PASEF) and the second was Parallel Accumulation-Serial Fragmentation combined with data-independent acquisition (diaPASEF) [2], [3]. Protein assembly was performed following search against the Swiss-Prot Human database using Peaks Studio for DDA datasets and Spectronaut for DIA datasets. The assembled result contains identified PSMs, peptides and proteins that are above threshold for each HeLa and SiHa sample. Coverage-wise, for DDA-PASEF, approximately 6,090 and 7,298 proteins were quantified for HeLa and SiHA sample, while13,339 and 8,773 proteins were quantified by diaPASEF for HeLa for SiHa sample, respectively. Consistency-wise, diaPASEF has fewer missing values (∼ 2%) compared to its DDA counterparts (∼5–7%). The mass spectrometry proteomics data have been deposited to the ProteomeXchange Consortium (http://proteomecentral.proteomexchange.org) via the iProX partner repository [4] with the dataset identifier PXD029773.

## Specifications Table

**Table:** 

Subject	*Biological science* Computational biology Proteomics
Specific subject area	Proteomics, bioinformatic, protein complexes, missing protein recovery
Type of data	Liquid chromatography tandem mass spectrometry (LC–MS/MS) data.
How the data were acquired	LC-MS/MS acquisition on a nanoElute LC system coupled to a timsTOF Pro-mass spectrometer.
Data format	Raw and processed.
Description of data collection	HeLa and SiHa human cell line digest were purchased from Thermo Scientific™ (Thermo Scientific, MA). The process of culturing and digesting can be found in the previous report [5]. For diaPASEF analysis, sample fractionation was performed. The samples were analyzed on a nanoElute UHPLC (Bruker Daltonics, Germany) coupled to a timsTOF Pro (Bruker Daltonics, Germany) equipped with a CaptiveSpray ion source, to obtain the proteomic information of HeLa and SiHa cells. Protein identification was done by performing library search against the reference library obtained from Swiss-Prot Human database (downloaded on May 8th, 2019) comprising 20,421 sequences in total. Peaks Studio (version 10.5) was used as search engine for DDA-PASEF analysis and Spectronaut (Biognosys, CH, Version 14.5.200813.47784) was used for diaPASEF analysis.
Data source location	School of Biological Sciences, Nanyang Technological University, 60 Nanyang Dr, Singapore. Zhejiang Provincial Laboratory of Life Sciences and Biomedicine, Key Laboratory of Structural Biology of Zhejiang Province, School of Life Sciences, Westlake University, Zhejiang, China.
Data accessibility	Repository name: ProteomeXchange Data identification number: PXD029773 Direct URL to data: http://proteomecentral.proteomexchange.org/cgi/GetDataset?ID=PXD029773
Related research article	W. Kong, B.J.H. Wong, H. Gao, T. Guo, X. Liu, X. Du, L. Wong, W.W.B. Goh PROTREC: A probability-based approach for recovering missing proteins based on biological networks Journal of Proteomics. 250 (2022) 104,392. https://doi.org/10.1016/j.jprot.2021.104392[1]

## Value of the Data

• This pairing of DDA and DIA data on the lateset PASEF technologies presents powerful analytical possibilities and allows scientists to compare and evaluate the previously acquired DDA or DIA non-PASEF data captured from HeLa and SiHa. This can assist in investigation into the added information value in terms of new observable proteins and data consistency given the PASEF technology.
• Researchers who are interested in the study of: mass spectrometry proteomics, protein identification and validation, protein assembly and missing protein recovery evaluation assembly, can benefit from these data.
• This dataset has replicate information in both HeLa and SiHa data, with three technical replicates in DDA data and DIA SiHa data, and two replicates in DIA HeLa data. This enables investigation into technical reproducibility on PASEF technology as well as possibility of using the data as benchmark to evaluate data quality given other proteomic platforms.
• This pairing of DDA-PASEF and diaPASEF data can be used to evaluate missing protein predictions. Given the logic that the DDA and DIA acquisition methods are performed along the same cell line, predictions made in the dirtier, but more accessible DDA platform can be verified on the more extensive DIA platform. Estimation of prediction accuracy can be obtained by comparing the prediction results with the DIA data.
• Conventional method which uses two-peptide rule to resolve protein assembly problems overlook the value of ambiguous protein and suffer from information loss. This dataset can also be used to investigate issues with protein assembly, especially on issues concerning the use of ambiguous peptide information considering the strength of PASEF on more identified peptides from HeLa and SiHa.

## 1 Data Description

• HeLa and SiHa dataset acquired using DDA-PASEF

The HeLa and SiHa DDA-PASEF datasets have only one phenotype class with three technical replicates. Both datasets were analyzed by Peaks Studio (Bioinformatics Solution Inc; version 10.5, April 14th, 2020) to search against the reference library obtained. The Swiss-Prot Human reference library (May 8th, 2019) contains 20,421 sequences.

**Fig. 1:**
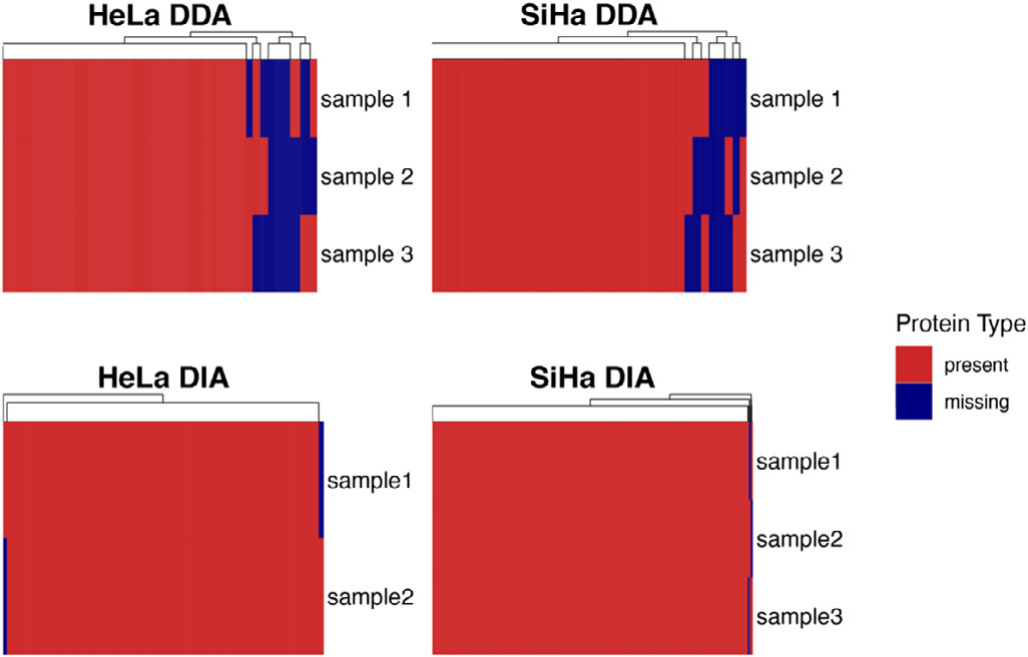
Heatmap of HeLa and SiHa DDA and DIA protein data. These four heatmaps show the completeness of protein information across four datasets. Red and blue areas denote protein “presence” and “missingness”, respectively. Each row corresponds to a sample. The top two heatmaps show the missing values in DDA datasets and have been published in the Supplementary Fig. 1 in the associated research article [1]. The bottom two heatmaps were added to this data article to show the distribution of missing values in the corresponding DIA datasets. The relatively higher coverage and consistency of the DIA dataset allows it to serve as a performance benchmark against any method developed on the DDA dataset.

For HeLa DDA-PASEF, approximately 310,277 PSMs, 57,856 peptides and 6090 proteins were identified across the three replicates at a 1% peptide FDR. For SiHa, approximately 351,782 PSMs, 74,658 peptides and 7298 proteins were identified across the three replicates at a 1% peptide FDR. Detailed information of DDA identification results can be found in Table 1. For protein data, there are 7% and 5% missing values in HeLa and SiHa, respectively, as shown in the top two heatmaps in Fig. 1.

• HeLa and SiHa dataset acquired using diaPASEF

**Table 1:** Summary table of DDA database search results.

HeLA DDA sample								
	Identified					#Proteins *		
HeLa DDA	#PSMs	#Scans	#Features **	#Peptides	#Sequences	Groups	All	Top
Total	310,277	307,568	132,883	57,856	56,621	5995	6598	6090
Sample 1	106,116	105,228	45,269	49,522	48,733	5604	6138	5681
Sample 2	103,006	102,077	44,232	48,464	47,606	5546	6092	5623
Sample 3	101,155	100,263	43,382	47,566	46,731	5574	6114	5662

SiHa DDA sample								

	Identified					#Proteins *		
SiHa DDA	#PSMs	#Scans	#Features **	#Peptides	#Sequences	Groups	All	Top

Total	351,782	348,848	177,581	74,658	73,046	7233	7833	7289
Sample 1	118,387	117,396	59,667	63,350	62,150	6855	7403	6912
Sample 2	116,149	115,180	58,701	63,011	61,828	6844	7389	6898
Sample 3	117,246	116,272	59,213	63,320	62,113	6824	7358	6883

These two tables show the total and sample individual number of identified PSMs, peptides and proteins in both HeLa DDA and SiHa DDA datasets. The * sign indicates that proteins with significance peptides are used in counts. The ** sign indicates that features are identified by DB search only.

The HeLa diaPASEF dataset contains two technical replicates while the SiHa diaPASEF dataset contains three technical replicates. To generate spectral library, HeLa and SiHa sample were first analyzed by timsTOF Pro-in DDA-PASEF mode, then the sample DDA data were performed library search against Swiss-Prot Human database by Spectronaut. The version of Spectronaut used is 14.5.200813.47784. The Swiss-Prot Human reference library contains 20,421 sequences and is downloaded on May 8th, 2019.

The project-specific HeLa library from 24 high-pH reversed-phase peptide fractions consisted of 301,353 target precursors and 16,578 target proteins on average. The project-specific SiHa library from 6 high-pH reversed-phase peptide fractions consisted of 153,771 target precursors and 9774 target proteins on average. For diaPASEF data analysis, HeLa and SiHa data was performed library search using respective library with Spectronaut (Biognosys, CH, version 14.5.200813.47784).

There are on average 9821 proteins quantified for HeLa DIA data and 8774 proteins for SiHa DIA data. In general, diaPASEF has higher consistency (∼2% data holes) and protein coverage compared to its DDA counterpart (∼5–7%) as shown in the bottom two heatmaps in Fig. 1.

## 2 Experimental Design, Materials and Methods

### 2.1 HeLa and SiHa data acquired by DDA-PASEF

#### 2.1.1 Sample collection

The HeLa and SiHa human cell line digests were purchased from Thermo Scientific™ (Thermo Scientific, MA). The process of culturing and digesting can be found in the previously published report [5]. Briefly, the cell line was cultured in Dulbecco's modified Eagle's medium with 10% fetal bovine serum, 20 mM glutamine and 1% penicillin-streptomycin. The cells were collected by centrifugation, washed once by phosphate-buffered saline, and then subsequently flash-frozen in liquid nitrogen before storing at −80 °C. The cells were subsequently subjected to lysis, reduction and alkylation according to previously published protocols [6]. The cells were treated in the lysis buffer with chloroacetamide (PreOmics) at a ratio of 1–5 µg protein per 1 µl lysis buffer. The lysates were then heated to 95 °C for 10 min and then sonicated to denature proteins, shear DNA and enhance cell disruption. Proteins were cleaved by enzymes overnight by adding equal amounts of LysC and trypsin in a 1:100 (wt/wt) enzyme:protein ratio. De-salting and purification were performed on a styrendivinylbenzene reversed-phase sulfonate (SDB-RPS) sorbent following the PreOmics iST protocol. Purified and dried peptide powder was reconstituted in buffer A (0.1% formic acid in water) for subsequent LC-MS analysis.

#### 2.1.2 Liquid chromatography mass spectrometry in DDA-PASEF mode

Liquid chromatography (LC) was performed on a nanoElute UHPLC (Bruker Daltonics, Germany). Around 200 ng peptides were separated within 120 min at a flowrate of 300nL/min on a commercially available reverse-phase C_18_ column with an integrated CaptiveSpray Emitter (25 cm x 75 µm ID, 1.6 µm, Aurora Series with CSI, IonOpticks, Australia) [3]. The separation temperature was kept by an integrated Toaster column oven at 50 °C. Mobile phases A and B were produced with 0.1 vol.-% formic acid in water and 0.1% formic acid in ACN.

The LC was coupled online to a hybrid timsTOF Pro (Bruker Daltonics, Germany) via a CaptiveSpray nano-electrospray ion source [3]. The timsTOF Pro-was operated in Data-Dependent Parallel Accumulation-Serial Fragmentation (PASEF) mode with 10 PASEF MS/MS frames in 1 complete frame. The capillary voltage was set to 1400 V, and the MS and MS/MS spectra were acquired from 100 to 1700 m/z. As for ion mobility range (1/K0), 0.6 to 1.6 Vs/cm^2^ for HeLa analysis and 0.7∼1.3 Vs/cm^2^ for SiHa analysis were used. The “target value” of 20,000 was applied to a repeated schedule, and the intensity threshold was set at 2500. The collision energy was ramped linearly as a function of mobility from 59 eV at 1/K0 = 1.6 Vs/cm^2^ to 20 eV at 1/K0 = 0.6 Vs/cm^2^.

#### 2.1.3 Library search of DDA data by Peaks Studio

The acquired HeLa and SiHa DDA spectra were processed using library search by Peaks Studio (Version10.5 build on April 15th, 2020, Bioinformatics Solution Inc.). The reference library is acquired from Swiss-Prot Human database which contains 20,421 sequences (May 8th, 2019). The raw data were analyzed with parent mass error tolerance set to 15 ppm and a fragment mass error tolerance of 0.05 Da. To account for post-translational modifications and chemical labelling, the following settings were used: Carbamidomethylation of cysteine residues was set as fixed modification, methionine oxidation and Acetylation (Protein N-term) was set as variable modification. Protein unique peptides was set to larger than 1 and a high confidence score of −10IgP >20 was applied to indicate an accurately identified protein. Detail parameters for DDA library search for both HeLa and SiHa can be found in Supplementary Table 1.

### 2.2 HeLa and SiHa data acquired by diaPASEF

#### 2.2.1 Sample collection

The diaPASEF analysis were performed on the same cell line as DDA-PASEF. Details of culturing and digesting process can be found in a previous report [5]. To generate a comprehensive library of precursor and fragment ions of HeLa and SiHa, peptide samples were fractionated at pH 10 with a ‘spider fractionator’ and then concatenated into 24 fractions for HeLa and 6 fractions for SiHa. The fractions were freeze-dried and re-constituted in 0.1% formic acid.

#### 2.2.2 Liquid chromatography mass spectrometry in diaPASEF mode

After high-pH reverse-phase fractionation, we employed a nanoElute liquid chromatography system (Bruker Daltonics) for peptide separation. Mobile phases A and B were with 0.1 vol.-% formic acid in water and 0.1% formic acid in ACN. For HeLa, the 24 high-pH reversed-phase fractions were separated within 100 min at a flowrate of 300 nL/min on a 25 cm analytical column. The fraction of B was increased from 2 to 22% in 90 min, 22 to 37% in 10 min, 37 to 80% in 10 min, and was sustained at 80% for 10 min. For SiHa, the 6 high-pH reverse-phase fractions were separated within 50 min at a flowrate of 300 nL/min on a 25 cm analytical column. The fraction of B was increased from 2 to 22% in 45 min, 22 to 37% in 5 min, 37 to 80% in 5 min, and was sustained at 80% for 5 min.

The LC was online with a timsTOF Pro (Bruker Daltonics, Germany), coupled with a CaptiveSpray nano-electrospray ion source. The dual TIMS analyzer was operated at a fixed duty cycle close to 100% by setting equal accumulation and ramp times at 100 ms each [3]. The Parallel Accumulation–Serial Fragmentation DDA method was used to select precursor ions for fragmentation, and the PASEF setting was one complete frame with 10 PASEF MS/MS frames for Hela and 4 PASEF MS/MS frames for SiHa analysis. The MS and MS/MS spectra were acquired between 100 and 1700 m/z, and an ion mobility range (1/K0) from 0.6 to 1.6 Vs/cm^2^ for Hela analysis and 0.7 to 1.3 Vs/cm^2^ for SiHa analysis was used. PASEF precursor selection of low m/z, singly charged ions was constrained by applying polygonal filtering. Precursors with 1–6 charges were selected with the target value set to 20,000 a.u. and intensity threshold to 2500 a.u.. To perform data-independent acquisition mode, the instrument control software was extended to define quadrupole isolation windows as a function of the TIMS scan time. Seamless and synchronous ramping of all applied voltage is achieved by modifying the instrument control electronics [3]. In Hela DIA experiment, we defined 25 Th isolation windows from m/z 400 to 1200 and totally 64 windows were defined. Similarly, we defined 28 Th isolation windows from m/z 384 to 1059 and totally 56 windows in the SiHa DIA experiment. For both scan modes, the collision energy was ramped linearly as a function of mobility from 59 eV at 1/K0 = 1.6 Vs/cm^2^ to 20 eV at 1/K0 = 0.6 Vs/cm^2^.

#### 2.2.3 Library search of DIA data by spectronaut

The library search of DIA data consists of two steps and is accomplished using Spectronaut (Biognosys, CH, Version 14.5.200813.47784). Firstly, HeLa and SiHa DDA data were analyzed in Spectronaut using a Pulsar search schema with default settings to generate respective spectral library. The calibration search was dynamic and MS1, MS2 correction factor was 1. Data were searched against the Swiss-Prot Human database (20,421 sequences, downloaded on May 8th, 2019), with trypsin as the protease. To account for post-translational modifications and chemical labelling settings, carbamidomethylation of cysteine residues was defined as a fixed modification, and methionine oxidation and acetylation of protein N-termini were defined as variable modifications. An FDR less than 1% was ensured for both the peptide spectrum match level and the protein level. DIA Library information is shown in Supplementary Table 2. Secondly, for DIA data mapping and analysis, HeLa and SiHa DIA data were performed library search against pre-generated respective spectral libraries using Spectronaut. Protein inference was performed via ID-Picker. The FDR was controlled at < 1% for both peptide precursors and assembled proteins. Detailed DIA data processing parameters with Spectronaut of HeLa and SiHa can be found in Supplementary Tables 3 and 4, respectively.

## CRediT authorship contribution statement

**Zelu Huang:** Writing – original draft, Visualization, Writing – review & editing. **Weijia Kong:** Conceptualization, Data curation, Visualization. **Bertrand Jernhan Wong:** Conceptualization, Data curation, Visualization. **Huanhuan Gao:** Software, Resources, Investigation. **Tiannan Guo:** Software, Resources, Investigation. **Xianming Liu:** Software, Resources, Investigation. **Xiaoxian Du:** Software, Resources, Investigation. **Limsoon Wong:** Conceptualization, Data curation, Visualization. **Wilson Wen Bin Goh:** Conceptualization, Supervision, Writing – review & editing.

## Declaration of Competing Interest

The authors declare that they have no known competing financial interests or personal relationships that could have appeared to influence the work reported in this paper.

## Appendix Supplementary materials

Image, application 1
